# P-778. Antimicrobial Effect of Nitric Oxide on Cystic Fibrosis Pathogens

**DOI:** 10.1093/ofid/ofae631.972

**Published:** 2025-01-29

**Authors:** Joy E Gibson, Christopher I Youssef, Michael N Neely

**Affiliations:** Children's Hospital Los Angeles, Los Angeles, California; Children's Hospital Los Angeles, Los Angeles, California; The Saban Research Institute, Children’s Hospital Los Angeles, University of Southern California, Los Angeles, CA, USA, Los Angeles, California

## Abstract

**Background:**

Despite highly effective modulator therapy, bacterial pathogens persist in the airways of children and adults with CF. *Staphylococcus aureus* (Sa) and *Pseudomonas aeruginosa* (Pa) are most commonly identified, but others like *Mycobacterium abscessus* complex (MABSC) and *Burkholderia cepacia* complex (Bcc) are particularly challenging due to the high frequency of intrinsic and acquired antibiotic resistance. Treatment options for MABSC are limited, requiring prolonged exposure to multiple antibiotics that may cause significant toxicity and frequent failure. Novel treatment approaches are a high priority for management of infections in people with CF. One potential adjunctive therapy is inhaled nitric oxide (NO), which is frequently used in ventilated patients and has been explored for treatment of several pulmonary infections, given its antimicrobial properties.

**Methods:**

In this study, we compared NO response in Sa, Pa, MABSC, and Bcc. We used chemical donors of NO, DETA NONOate and PAPA NONOate, to provide continuous or intermittent exposure, respectively, and compared growth curves assessed by OD_600_ over 24 hours for Sa, Pa, and Bcc, and 7 days for MABSC. We also exposed MABSC to different concentrations and frequencies of DETA and PAPA NONOate to determine the pharmacodynamics of this effect. To better correlate with clinical use, we further compared growth inhibition with gaseous NO delivered at 160 ppm thrice daily.

**Results:**

We found that treatment with NONOate resulted in near complete inhibition of growth in MABSC at all concentrations, dose-dependent inhibition of Pa and Bcc, with a more potent affect on Bcc growth, and minimal growth inhibition of Sa. We quantified the minimum inhibitory concentration (MIC) of DETA NONOate in MABSC to be 660 µM and found the effect of NO on MABSC to be time-dependent based on linear regression of delta CFU relative to time above MIC (R^2^ =0.848), peak to MIC (R^2^ =0.344), and area under the curve to MIC (R^2^ =0.745). Gaseous NO resulted in greater than 50% growth inhibition of MABSC, but no inhibition in Pa or Bcc.

**Conclusion:**

These findings indicate that inhaled NO may be a promising adjunctive therapy for treatment of MABSC, but may be less effective against other CF pathogens.

**Disclosures:**

**All Authors**: No reported disclosures

